# The chalcone ethyl 2-[4-(2-{2,4-bis­[(eth­oxy­carbon­yl)meth­oxy]benzo­yl}ethen­yl)phen­oxy]acetate

**DOI:** 10.1107/S1600536813033114

**Published:** 2013-12-14

**Authors:** Xiaofeng Liu

**Affiliations:** a School of Chemistry & Environmental Engineering, Jiujiang University, Jiujiang 332005, People’s Republic of China

## Abstract

In the title mol­ecule, C_27_H_30_O_10_, the benzene rings form a dihedral angle of 14.9 (2)°. The C=C bond is in an *E* conformation. In the crystal, weak C—H⋯O hydrogen bonds connect the mol­ecules, forming a three-dimensional network.

## Related literature   

For a related structure, see: Wang *et al.* (2011 ▶).
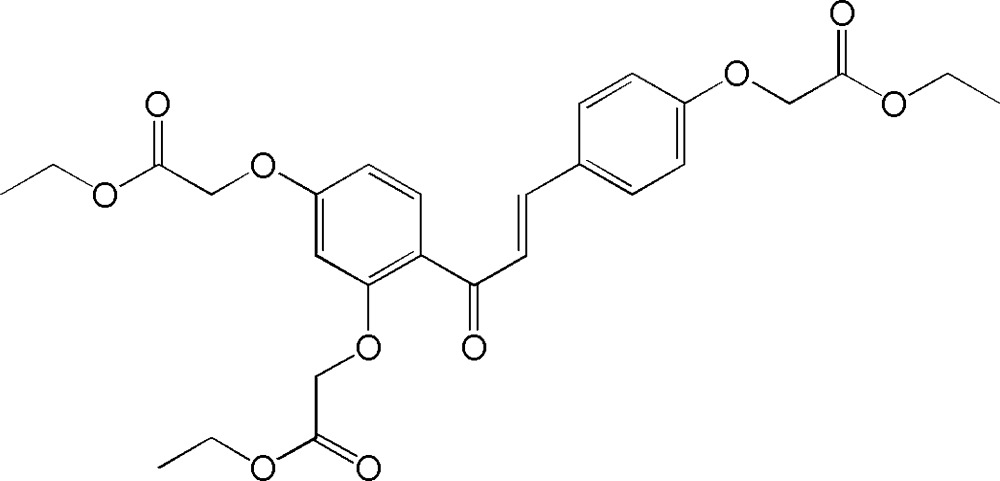


## Experimental   

### 

#### Crystal data  

C_27_H_30_O_10_
*M* *_r_* = 514.51
Triclinic, 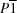

*a* = 7.8225 (16) Å
*b* = 13.579 (3) Å
*c* = 13.780 (3) Å
α = 63.004 (3)°
β = 87.157 (4)°
γ = 83.377 (4)°
*V* = 1295.6 (5) Å^3^
*Z* = 2
Mo *K*α radiation
μ = 0.10 mm^−1^
*T* = 298 K
0.12 × 0.10 × 0.10 mm

#### Data collection  

Bruker APEXII CCD diffractometer
Absorption correction: multi-scan (*SADABS*; Sheldrick, 1996 ▶) *T*_min_ = 0.988, *T*_max_ = 0.990
16520 measured reflections
4538 independent reflections
4137 reflections with *I* > 2σ(*I*)
*R*_int_ = 0.018

#### Refinement  

*R*[*F* ^2^ > 2σ(*F* ^2^)] = 0.075
*wR*(*F* ^2^) = 0.178
*S* = 1.20
4538 reflections
337 parameters
H-atom parameters constrained
Δρ_max_ = 0.39 e Å^−3^
Δρ_min_ = −0.23 e Å^−3^

### 

Data collection: *APEX2* (Bruker, 2004 ▶); cell refinement: *SAINT* (Bruker, 2004 ▶); data reduction: *SAINT*; program(s) used to solve structure: *SHELXS97* (Sheldrick, 2008 ▶); program(s) used to refine structure: *SHELXL97* (Sheldrick, 2008 ▶); molecular graphics: *PLATON* (Spek, 2009 ▶); software used to prepare material for publication: *SHELXTL* (Sheldrick, 2008 ▶).

## Supplementary Material

Crystal structure: contains datablock(s) I, New_Global_Publ_Block. DOI: 10.1107/S1600536813033114/lh5675sup1.cif

Structure factors: contains datablock(s) I. DOI: 10.1107/S1600536813033114/lh5675Isup2.hkl

Supporting information file. DOI: 10.1107/S1600536813033114/lh5675Isup3.cml

Additional supporting information:  crystallographic information; 3D view; checkCIF report

## Figures and Tables

**Table 1:** Hydrogen-bond geometry (Å, °)

*D*—H⋯*A*	*D*—H	H⋯*A*	*D*⋯*A*	*D*—H⋯*A*
C3—H3⋯O1^i^	0.93	2.52	3.276 (4)	138
C16—H16*B*⋯O7^ii^	0.97	2.58	3.337 (4)	135
C20—H20*B*⋯O4^ii^	0.97	2.60	3.360 (4)	136
C24—H24*B*⋯O3^iii^	0.97	2.53	3.339 (4)	141
C27—H27*B*⋯O6^iv^	0.96	2.56	3.426 (4)	150

Symmetry codes: (i) 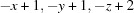; (ii) 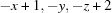; (iii) 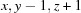; (iv) 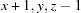.

**Fig. 1.**
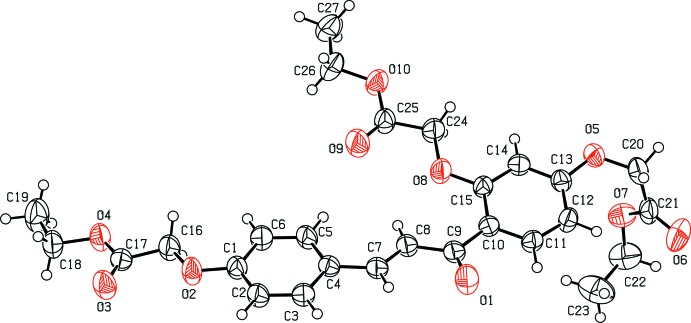
View of a molecule of (I) showing displacement ellipsoids drawn at the 50% probability level.

**Fig. 2.**
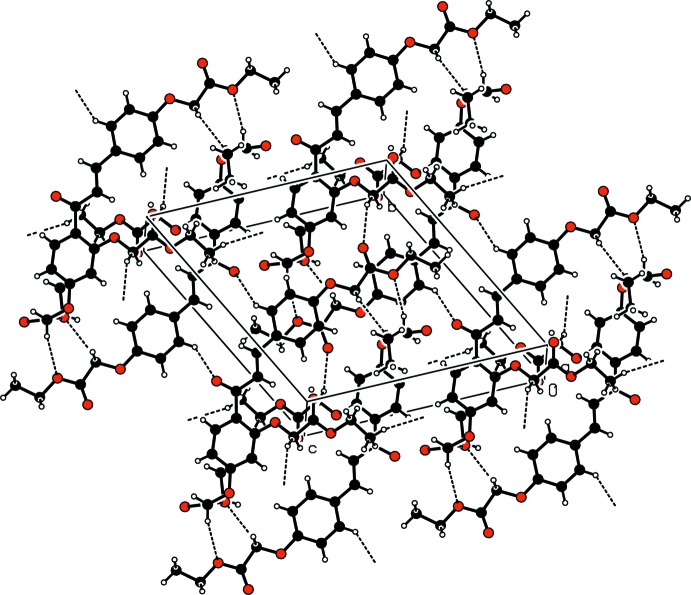
The crystal packing for (I), with weak C—H···O interactions shown as dashed lines.

## supplementary crystallographic information

### 1. Comment

It has been determined in the authors lab that the title compound is a potential inhibitor of mushroom tyrosinase (results to be published soon) and the crystal structure determination of the title compound has been carried out to investigate its structure property relationships.

In the title molecule (I, Fig. 1) the two benzene rings (C10-C15/C1-C6) form a dihedral angle of 14.9 (2)°. The C═C bond is in an E conformation. In the crystal, weak C—H···O hydrogen bonds connect molecules forming a three-dimensional network (Fig. 2). The structure of a related compound, (E)-1-(2,4-Dihydroxyphenyl)-3-(4-hydroxyphenyl)prop-2-en-1-one monohydrate, has been published previously (Wang *et al.*, 2011).

### 2. Experimental

Isoliquiritigenin (2.56 g, 0.01 mol) and potassium hydroxide (2.45 g, 0.0438 mol) were dissolved in dry acetone (100 ml) in a three-neck flask. Then ethyl bromoacetate (6.93 g, 0.0415 mol) was dropwise added at room temperature and vigorously stirred for 3 h. The progress of the reaction was monitored by TCL (Si gel, developing solvent *V* (ethyl acetate) /*V* (benzene) = 1:2). After suction filtration and distilled to remove the solvent, a light yellow solid was obtained, 4.39 g, yield 85.4%. Recrystallization from ethanol produced light yellow needles.

### 3. Refinement

All hydrogen atoms were placed in ideal positions with the C—H = 0.93 Å, C—H = 0.96 Å and C—H=0.97 Å with *U*_iso_(H) = 1.2*U*_eq_(C) or 1.5*U*_eq_(C_metyhl_).

### Crystal data

**Table:**

C_27_H_30_O_10_	*Z* = 2
*M**_r_* = 514.51	*F*(000) = 544
Triclinic, *P*1	*D*_x_ = 1.319 Mg m^−^^3^
Hall symbol: -P 1	Mo *K*α radiation, λ = 0.71073 Å
*a* = 7.8225 (16) Å	Cell parameters from 2402 reflections
*b* = 13.579 (3) Å	θ = 0.0–0.0°
*c* = 13.780 (3) Å	µ = 0.10 mm^−^^1^
α = 63.004 (3)°	*T* = 298 K
β = 87.157 (4)°	Block, light yellow
γ = 83.377 (4)°	0.12 × 0.10 × 0.10 mm
*V* = 1295.6 (5) Å^3^	

### Data collection

**Table:**

Bruker APEXII CCD diffractometer	4538 independent reflections
Radiation source: fine-focus sealed tube	4137 reflections with *I* > 2σ(*I*)
Graphite monochromator	*R*_int_ = 0.018
φ and ω scans	θ_max_ = 25.0°, θ_min_ = 1.7°
Absorption correction: multi-scan (*SADABS*; Sheldrick, 1996)	*h* = −7→9
*T*_min_ = 0.988, *T*_max_ = 0.990	*k* = −16→16
16520 measured reflections	*l* = −16→16

### Refinement

**Table:**

Refinement on *F*^2^	Primary atom site location: structure-invariant direct methods
Least-squares matrix: full	Secondary atom site location: difference Fourier map
*R*[*F*^2^ > 2σ(*F*^2^)] = 0.075	Hydrogen site location: inferred from neighbouring sites
*wR*(*F*^2^) = 0.178	H-atom parameters constrained
*S* = 1.20	*w* = 1/[σ^2^(*F*_o_^2^) + (0.0569*P*)^2^ + 1.0739*P*] where *P* = (*F*_o_^2^ + 2*F*_c_^2^)/3
4538 reflections	(Δ/σ)_max_ = 0.001
337 parameters	Δρ_max_ = 0.39 e Å^−^^3^
0 restraints	Δρ_min_ = −0.23 e Å^−^^3^

### Special details

**Table:**

Geometry. All e.s.d.'s (except the e.s.d. in the dihedral angle between two l.s. planes) are estimated using the full covariance matrix. The cell e.s.d.'s are taken into account individually in the estimation of e.s.d.'s in distances, angles and torsion angles; correlations between e.s.d.'s in cell parameters are only used when they are defined by crystal symmetry. An approximate (isotropic) treatment of cell e.s.d.'s is used for estimating e.s.d.'s involving l.s. planes.
Refinement. Refinement of *F*^2^ against ALL reflections. The weighted *R*-factor *wR* and goodness of fit *S* are based on *F*^2^, conventional *R*-factors *R* are based on *F*, with *F* set to zero for negative *F*^2^. The threshold expression of *F*^2^ > σ(*F*^2^) is used only for calculating *R*-factors(gt) *etc*. and is not relevant to the choice of reflections for refinement. *R*-factors based on *F*^2^ are statistically about twice as large as those based on *F*, and *R*- factors based on ALL data will be even larger.

### Fractional atomic coordinates and isotropic or equivalent isotropic displacement parameters (Å^2^)

**Table:**

	*x*	*y*	*z*	*U*_iso_*/*U*_eq_	
C1	0.7304 (4)	0.4831 (2)	0.6468 (2)	0.0502 (7)	
C2	0.7010 (4)	0.5453 (2)	0.7028 (2)	0.0549 (8)	
H2	0.7100	0.6213	0.6670	0.066*	
C3	0.6586 (4)	0.4962 (2)	0.8106 (2)	0.0532 (8)	
H3	0.6387	0.5397	0.8471	0.064*	
C4	0.6444 (4)	0.3821 (2)	0.8675 (2)	0.0456 (7)	
C5	0.6802 (4)	0.3199 (2)	0.8103 (2)	0.0525 (7)	
H5	0.6765	0.2434	0.8467	0.063*	
C6	0.7211 (5)	0.3687 (2)	0.7015 (3)	0.0570 (8)	
H6	0.7423	0.3258	0.6646	0.068*	
C7	0.5925 (4)	0.3344 (2)	0.9813 (2)	0.0478 (7)	
H7	0.5790	0.3829	1.0124	0.057*	
C8	0.5619 (4)	0.2298 (2)	1.0465 (2)	0.0467 (7)	
H8	0.5751	0.1776	1.0197	0.056*	
C9	0.5081 (4)	0.1960 (2)	1.1589 (2)	0.0475 (7)	
C10	0.4790 (4)	0.0787 (2)	1.2360 (2)	0.0415 (6)	
C11	0.3962 (4)	0.0624 (2)	1.3330 (2)	0.0459 (7)	
H11	0.3563	0.1246	1.3425	0.055*	
C12	0.3700 (4)	−0.0400 (2)	1.4151 (2)	0.0485 (7)	
H12	0.3136	−0.0470	1.4786	0.058*	
C13	0.4293 (4)	−0.1333 (2)	1.4016 (2)	0.0435 (6)	
C14	0.5085 (4)	−0.1219 (2)	1.3063 (2)	0.0463 (7)	
H14	0.5449	−0.1848	1.2970	0.056*	
C15	0.5343 (4)	−0.0178 (2)	1.2239 (2)	0.0432 (6)	
C16	0.7769 (5)	0.4821 (2)	0.4757 (2)	0.0587 (8)	
H16A	0.6704	0.4490	0.4833	0.070*	
H16B	0.8709	0.4230	0.5006	0.070*	
C17	0.8034 (4)	0.5619 (2)	0.3588 (2)	0.0517 (7)	
C18	0.8388 (5)	0.5709 (3)	0.1834 (2)	0.0623 (9)	
H18A	0.9452	0.6053	0.1695	0.075*	
H18B	0.7445	0.6287	0.1514	0.075*	
C19	0.8467 (6)	0.4911 (3)	0.1360 (3)	0.0855 (12)	
H19A	0.9402	0.4342	0.1687	0.128*	
H19B	0.8644	0.5300	0.0589	0.128*	
H19C	0.7405	0.4579	0.1501	0.128*	
C20	0.3347 (4)	−0.2565 (3)	1.5781 (2)	0.0563 (8)	
H20A	0.3860	−0.2134	1.6068	0.068*	
H20B	0.3559	−0.3343	1.6303	0.068*	
C21	0.1446 (5)	−0.2243 (2)	1.5680 (2)	0.0547 (8)	
C22	−0.0998 (5)	−0.1807 (3)	1.4570 (4)	0.0775 (11)	
H22A	−0.1640	−0.1962	1.5235	0.093*	
H22B	−0.1500	−0.2159	1.4194	0.093*	
C23	−0.1101 (6)	−0.0602 (3)	1.3875 (4)	0.0955 (14)	
H23A	−0.0536	−0.0262	1.4231	0.143*	
H23B	−0.2287	−0.0300	1.3748	0.143*	
H23C	−0.0548	−0.0455	1.3192	0.143*	
C24	0.6858 (5)	−0.0966 (2)	1.1165 (3)	0.0592 (8)	
H24A	0.5934	−0.1392	1.1180	0.071*	
H24B	0.7663	−0.1441	1.1747	0.071*	
C25	0.7749 (4)	−0.0555 (3)	1.0089 (2)	0.0522 (7)	
C26	0.9388 (5)	−0.1206 (3)	0.8969 (3)	0.0672 (9)	
H26A	1.0458	−0.0924	0.8998	0.081*	
H26B	0.8735	−0.0652	0.8340	0.081*	
C27	0.9746 (5)	−0.2257 (3)	0.8881 (3)	0.0744 (10)	
H27A	1.0383	−0.2802	0.9509	0.112*	
H27B	1.0409	−0.2128	0.8236	0.112*	
H27C	0.8680	−0.2523	0.8839	0.112*	
O1	0.4841 (4)	0.26559 (18)	1.19312 (19)	0.0826 (9)	
O2	0.7698 (3)	0.54015 (16)	0.53900 (17)	0.0655 (7)	
O3	0.8152 (4)	0.65850 (18)	0.32433 (19)	0.0776 (8)	
O4	0.8129 (3)	0.50759 (17)	0.29960 (16)	0.0608 (6)	
O5	0.4174 (3)	−0.24062 (16)	1.47859 (16)	0.0541 (5)	
O6	0.0654 (4)	−0.2021 (3)	1.6330 (2)	0.0971 (10)	
O7	0.0789 (3)	−0.22501 (18)	1.48300 (19)	0.0618 (6)	
O8	0.6192 (3)	−0.00224 (16)	1.13008 (17)	0.0653 (7)	
O9	0.7865 (4)	0.03736 (19)	0.9457 (2)	0.0800 (8)	
O10	0.8417 (3)	−0.14310 (17)	0.99470 (18)	0.0649 (6)	

### Atomic displacement parameters (Å^2^)

**Table:**

	*U*^11^	*U*^22^	*U*^33^	*U*^12^	*U*^13^	*U*^23^
C1	0.0668 (19)	0.0382 (15)	0.0429 (15)	−0.0079 (13)	0.0095 (13)	−0.0164 (13)
C2	0.084 (2)	0.0296 (14)	0.0496 (17)	−0.0093 (14)	0.0116 (15)	−0.0164 (13)
C3	0.077 (2)	0.0357 (15)	0.0514 (17)	−0.0058 (14)	0.0095 (15)	−0.0243 (14)
C4	0.0550 (17)	0.0351 (14)	0.0448 (15)	−0.0043 (12)	0.0028 (13)	−0.0167 (12)
C5	0.077 (2)	0.0294 (14)	0.0467 (16)	−0.0097 (13)	0.0093 (14)	−0.0137 (12)
C6	0.087 (2)	0.0378 (15)	0.0510 (17)	−0.0099 (15)	0.0129 (16)	−0.0245 (14)
C7	0.0630 (18)	0.0347 (14)	0.0464 (16)	−0.0035 (13)	0.0047 (13)	−0.0198 (13)
C8	0.0611 (18)	0.0381 (15)	0.0424 (15)	−0.0054 (13)	0.0022 (13)	−0.0195 (12)
C9	0.0638 (19)	0.0367 (14)	0.0436 (15)	−0.0035 (13)	0.0021 (13)	−0.0202 (13)
C10	0.0482 (16)	0.0381 (14)	0.0398 (14)	−0.0039 (11)	0.0011 (12)	−0.0191 (12)
C11	0.0592 (18)	0.0397 (15)	0.0457 (15)	−0.0066 (13)	0.0067 (13)	−0.0257 (13)
C12	0.0607 (18)	0.0477 (16)	0.0422 (15)	−0.0140 (13)	0.0145 (13)	−0.0242 (13)
C13	0.0474 (16)	0.0383 (14)	0.0421 (15)	−0.0092 (12)	0.0025 (12)	−0.0150 (12)
C14	0.0580 (18)	0.0355 (14)	0.0486 (16)	−0.0049 (12)	0.0045 (13)	−0.0222 (13)
C15	0.0545 (17)	0.0392 (14)	0.0379 (14)	−0.0053 (12)	0.0060 (12)	−0.0196 (12)
C16	0.088 (2)	0.0410 (16)	0.0473 (17)	−0.0109 (15)	0.0127 (16)	−0.0206 (14)
C17	0.067 (2)	0.0381 (16)	0.0475 (16)	−0.0028 (13)	0.0083 (14)	−0.0183 (13)
C18	0.085 (2)	0.0570 (19)	0.0402 (16)	−0.0134 (17)	0.0100 (15)	−0.0179 (15)
C19	0.126 (4)	0.080 (3)	0.057 (2)	−0.022 (2)	0.017 (2)	−0.037 (2)
C20	0.077 (2)	0.0471 (17)	0.0390 (15)	−0.0150 (15)	0.0043 (15)	−0.0130 (13)
C21	0.079 (2)	0.0411 (16)	0.0405 (16)	−0.0178 (15)	0.0179 (15)	−0.0143 (13)
C22	0.066 (2)	0.069 (2)	0.105 (3)	−0.0102 (18)	−0.001 (2)	−0.045 (2)
C23	0.076 (3)	0.072 (3)	0.122 (4)	−0.001 (2)	−0.025 (3)	−0.028 (3)
C24	0.086 (2)	0.0410 (16)	0.0536 (18)	−0.0068 (15)	0.0161 (16)	−0.0254 (14)
C25	0.070 (2)	0.0463 (17)	0.0440 (16)	−0.0119 (14)	0.0091 (14)	−0.0231 (14)
C26	0.071 (2)	0.081 (2)	0.063 (2)	−0.0145 (18)	0.0210 (17)	−0.0441 (19)
C27	0.080 (3)	0.085 (3)	0.071 (2)	0.000 (2)	0.0112 (19)	−0.049 (2)
O1	0.149 (3)	0.0424 (12)	0.0593 (14)	−0.0150 (14)	0.0280 (15)	−0.0274 (11)
O2	0.1146 (19)	0.0361 (11)	0.0445 (12)	−0.0161 (11)	0.0222 (12)	−0.0174 (9)
O3	0.133 (2)	0.0401 (13)	0.0555 (14)	−0.0114 (13)	0.0162 (14)	−0.0190 (11)
O4	0.0943 (17)	0.0451 (12)	0.0437 (11)	−0.0165 (11)	0.0133 (11)	−0.0197 (10)
O5	0.0667 (14)	0.0383 (11)	0.0483 (12)	−0.0082 (9)	0.0106 (10)	−0.0121 (9)
O6	0.114 (2)	0.114 (2)	0.0670 (17)	−0.0088 (18)	0.0327 (16)	−0.0479 (17)
O7	0.0630 (14)	0.0582 (13)	0.0735 (15)	−0.0026 (11)	−0.0009 (11)	−0.0387 (12)
O8	0.1049 (18)	0.0377 (11)	0.0509 (12)	−0.0062 (11)	0.0263 (12)	−0.0208 (10)
O9	0.124 (2)	0.0459 (14)	0.0626 (15)	−0.0082 (13)	0.0233 (15)	−0.0203 (12)
O10	0.0907 (17)	0.0486 (12)	0.0585 (13)	−0.0094 (11)	0.0247 (12)	−0.0288 (11)

### Geometric parameters (Å, º)

**Table:**

C1—O2	1.366 (3)	C17—O4	1.319 (4)
C1—C2	1.375 (4)	C18—O4	1.450 (4)
C1—C6	1.394 (4)	C18—C19	1.492 (5)
C2—C3	1.365 (4)	C18—H18A	0.9700
C2—H2	0.9300	C18—H18B	0.9700
C3—C4	1.399 (4)	C19—H19A	0.9600
C3—H3	0.9300	C19—H19B	0.9600
C4—C5	1.393 (4)	C19—H19C	0.9600
C4—C7	1.455 (4)	C20—O5	1.422 (4)
C5—C6	1.375 (4)	C20—C21	1.496 (5)
C5—H5	0.9300	C20—H20A	0.9700
C6—H6	0.9300	C20—H20B	0.9700
C7—C8	1.335 (4)	C21—O6	1.193 (4)
C7—H7	0.9300	C21—O7	1.307 (4)
C8—C9	1.457 (4)	C22—O7	1.451 (4)
C8—H8	0.9300	C22—C23	1.468 (5)
C9—O1	1.227 (3)	C22—H22A	0.9700
C9—C10	1.497 (4)	C22—H22B	0.9700
C10—C11	1.389 (4)	C23—H23A	0.9600
C10—C15	1.412 (4)	C23—H23B	0.9600
C11—C12	1.368 (4)	C23—H23C	0.9600
C11—H11	0.9300	C24—O8	1.415 (3)
C12—C13	1.387 (4)	C24—C25	1.494 (4)
C12—H12	0.9300	C24—H24A	0.9700
C13—O5	1.370 (3)	C24—H24B	0.9700
C13—C14	1.375 (4)	C25—O9	1.174 (4)
C14—C15	1.384 (4)	C25—O10	1.335 (4)
C14—H14	0.9300	C26—O10	1.437 (4)
C15—O8	1.363 (3)	C26—C27	1.481 (5)
C16—O2	1.412 (3)	C26—H26A	0.9700
C16—C17	1.499 (4)	C26—H26B	0.9700
C16—H16A	0.9700	C27—H27A	0.9600
C16—H16B	0.9700	C27—H27B	0.9600
C17—O3	1.189 (3)	C27—H27C	0.9600
			
O2—C1—C2	116.0 (2)	O4—C18—H18B	110.3
O2—C1—C6	124.5 (3)	C19—C18—H18B	110.3
C2—C1—C6	119.5 (3)	H18A—C18—H18B	108.6
C3—C2—C1	120.5 (3)	C18—C19—H19A	109.5
C3—C2—H2	119.8	C18—C19—H19B	109.5
C1—C2—H2	119.8	H19A—C19—H19B	109.5
C2—C3—C4	121.5 (3)	C18—C19—H19C	109.5
C2—C3—H3	119.2	H19A—C19—H19C	109.5
C4—C3—H3	119.2	H19B—C19—H19C	109.5
C5—C4—C3	117.1 (3)	O5—C20—C21	114.2 (3)
C5—C4—C7	123.4 (2)	O5—C20—H20A	108.7
C3—C4—C7	119.5 (2)	C21—C20—H20A	108.7
C6—C5—C4	121.7 (3)	O5—C20—H20B	108.7
C6—C5—H5	119.1	C21—C20—H20B	108.7
C4—C5—H5	119.1	H20A—C20—H20B	107.6
C5—C6—C1	119.6 (3)	O6—C21—O7	125.4 (4)
C5—C6—H6	120.2	O6—C21—C20	121.9 (3)
C1—C6—H6	120.2	O7—C21—C20	112.7 (3)
C8—C7—C4	128.1 (3)	O7—C22—C23	109.5 (3)
C8—C7—H7	116.0	O7—C22—H22A	109.8
C4—C7—H7	116.0	C23—C22—H22A	109.8
C7—C8—C9	121.0 (3)	O7—C22—H22B	109.8
C7—C8—H8	119.5	C23—C22—H22B	109.8
C9—C8—H8	119.5	H22A—C22—H22B	108.2
O1—C9—C8	119.6 (3)	C22—C23—H23A	109.5
O1—C9—C10	117.6 (3)	C22—C23—H23B	109.5
C8—C9—C10	122.8 (2)	H23A—C23—H23B	109.5
C11—C10—C15	116.3 (2)	C22—C23—H23C	109.5
C11—C10—C9	116.4 (2)	H23A—C23—H23C	109.5
C15—C10—C9	127.2 (2)	H23B—C23—H23C	109.5
C12—C11—C10	123.8 (3)	O8—C24—C25	107.2 (2)
C12—C11—H11	118.1	O8—C24—H24A	110.3
C10—C11—H11	118.1	C25—C24—H24A	110.3
C11—C12—C13	118.5 (3)	O8—C24—H24B	110.3
C11—C12—H12	120.7	C25—C24—H24B	110.3
C13—C12—H12	120.7	H24A—C24—H24B	108.5
O5—C13—C14	115.1 (2)	O9—C25—O10	124.3 (3)
O5—C13—C12	124.6 (2)	O9—C25—C24	127.2 (3)
C14—C13—C12	120.2 (2)	O10—C25—C24	108.5 (2)
C13—C14—C15	120.6 (2)	O10—C26—C27	108.1 (3)
C13—C14—H14	119.7	O10—C26—H26A	110.1
C15—C14—H14	119.7	C27—C26—H26A	110.1
O8—C15—C14	122.9 (2)	O10—C26—H26B	110.1
O8—C15—C10	116.4 (2)	C27—C26—H26B	110.1
C14—C15—C10	120.6 (2)	H26A—C26—H26B	108.4
O2—C16—C17	108.9 (2)	C26—C27—H27A	109.5
O2—C16—H16A	109.9	C26—C27—H27B	109.5
C17—C16—H16A	109.9	H27A—C27—H27B	109.5
O2—C16—H16B	109.9	C26—C27—H27C	109.5
C17—C16—H16B	109.9	H27A—C27—H27C	109.5
H16A—C16—H16B	108.3	H27B—C27—H27C	109.5
O3—C17—O4	125.1 (3)	C1—O2—C16	117.3 (2)
O3—C17—C16	125.8 (3)	C17—O4—C18	117.4 (2)
O4—C17—C16	109.1 (2)	C13—O5—C20	117.2 (2)
O4—C18—C19	106.9 (3)	C21—O7—C22	117.3 (3)
O4—C18—H18A	110.3	C15—O8—C24	118.7 (2)
C19—C18—H18A	110.3	C25—O10—C26	116.9 (3)
			
O2—C1—C2—C3	179.2 (3)	C11—C10—C15—O8	−178.5 (3)
C6—C1—C2—C3	−1.7 (5)	C9—C10—C15—O8	−2.1 (4)
C1—C2—C3—C4	0.3 (5)	C11—C10—C15—C14	−1.1 (4)
C2—C3—C4—C5	1.8 (5)	C9—C10—C15—C14	175.2 (3)
C2—C3—C4—C7	−177.7 (3)	O2—C16—C17—O3	−0.6 (5)
C3—C4—C5—C6	−2.6 (5)	O2—C16—C17—O4	179.0 (3)
C7—C4—C5—C6	176.9 (3)	O5—C20—C21—O6	−158.1 (3)
C4—C5—C6—C1	1.3 (5)	O5—C20—C21—O7	22.8 (4)
O2—C1—C6—C5	180.0 (3)	O8—C24—C25—O9	−1.1 (5)
C2—C1—C6—C5	0.9 (5)	O8—C24—C25—O10	179.0 (3)
C5—C4—C7—C8	−3.9 (5)	C2—C1—O2—C16	−171.3 (3)
C3—C4—C7—C8	175.5 (3)	C6—C1—O2—C16	9.6 (5)
C4—C7—C8—C9	−179.1 (3)	C17—C16—O2—C1	174.2 (3)
C7—C8—C9—O1	2.8 (5)	O3—C17—O4—C18	−0.3 (5)
C7—C8—C9—C10	−177.6 (3)	C16—C17—O4—C18	−179.9 (3)
O1—C9—C10—C11	11.9 (4)	C19—C18—O4—C17	179.4 (3)
C8—C9—C10—C11	−167.7 (3)	C14—C13—O5—C20	−179.2 (3)
O1—C9—C10—C15	−164.5 (3)	C12—C13—O5—C20	1.5 (4)
C8—C9—C10—C15	15.9 (5)	C21—C20—O5—C13	70.3 (3)
C15—C10—C11—C12	1.3 (4)	O6—C21—O7—C22	9.1 (5)
C9—C10—C11—C12	−175.5 (3)	C20—C21—O7—C22	−171.9 (3)
C10—C11—C12—C13	0.1 (5)	C23—C22—O7—C21	87.1 (4)
C11—C12—C13—O5	177.5 (3)	C14—C15—O8—C24	−0.8 (5)
C11—C12—C13—C14	−1.7 (4)	C10—C15—O8—C24	176.5 (3)
O5—C13—C14—C15	−177.4 (3)	C25—C24—O8—C15	−178.7 (3)
C12—C13—C14—C15	1.9 (4)	O9—C25—O10—C26	2.3 (5)
C13—C14—C15—O8	176.8 (3)	C24—C25—O10—C26	−177.7 (3)
C13—C14—C15—C10	−0.4 (4)	C27—C26—O10—C25	−170.4 (3)

### Hydrogen-bond geometry (Å, º)

**Table:**

*D*—H···*A*	*D*—H	H···*A*	*D*···*A*	*D*—H···*A*
C3—H3···O1^i^	0.93	2.52	3.276 (4)	138
C16—H16*B*···O7^ii^	0.97	2.58	3.337 (4)	135
C20—H20*B*···O4^ii^	0.97	2.60	3.360 (4)	136
C24—H24*B*···O3^iii^	0.97	2.53	3.339 (4)	141
C27—H27*B*···O6^iv^	0.96	2.56	3.426 (4)	150

Symmetry codes: (i) −*x*+1, −*y*+1, −*z*+2; (ii) −*x*+1, −*y*, −*z*+2; (iii) *x*, *y*−1, *z*+1; (iv) *x*+1, *y*, *z*−1.
